# Assessment of Cell-Cycle Arrest Biomarkers to Predict Early and Delayed Acute Kidney Injury

**DOI:** 10.1155/2015/158658

**Published:** 2015-03-18

**Authors:** Max Bell, Anders Larsson, Per Venge, Rinaldo Bellomo, Johan Mårtensson

**Affiliations:** ^1^Section of Anaesthesia and Intensive Care Medicine, Department of Physiology and Pharmacology, Karolinska Institutet, 17176 Stockholm, Sweden; ^2^Clinical Chemistry, Department of Medical Sciences, Uppsala University, 751 85 Uppsala, Sweden; ^3^Department of Intensive Care, Austin Hospital, Heidelberg, Melbourne, VIC 3084, Australia; ^4^Australian and New Zealand Intensive Care Research Centre, School of Preventive Medicine and Public Health, Monash University, Melbourne, VIC 3800, Australia

## Abstract

*Purpose*. To assess urinary tissue inhibitor of metalloproteinases-2 and insulin-like growth factor binding protein 7 ([TIMP-2]·[IGFBP7]), urinary neutrophil gelatinase-associated lipocalin (NGAL), and urinary cystatin-C as acute kidney injury predictors (AKI) exploring the association of nonrenal factors with elevated biomarker levels.* Methods*. We studied 94 patients with urine collected within 48 hours of ICU admission and no AKI at sampling. AKI was defined by the Kidney Disease: Improving Global Outcomes criteria. Predictive performance was assessed by the area under the receiver operating characteristics (ROC) curve. Associations between biomarkers and clinical factors were assessed by multivariate linear regression.* Results*. Overall, 19 patients (20%) developed AKI within 48 hours. [TIMP-2]·[IGFBP7], NGAL, or cystatin-C admission levels did not differ between patients without AKI and patients developing AKI. [TIMP-2]·[IGFBP7], NGAL, and cystatin-C were poor AKI predictors (ROC areas 0.34–0.51). Diabetes was independently associated with higher [TIMP-2]·[IGFBP7] levels (*P* = 0.02) but AKI was not (*P* = 0.24). Sepsis was independently associated with higher NGAL (*P* < 0.001) and cystatin-C (*P* = 0.003) levels.* Conclusions*. Urinary [TIMP-2]·[IGFBP7], NGAL, and cystatin-C should be used cautiously as AKI predictors in general ICU patients since urine levels of these biomarkers are affected by factors other than AKI and their performance can be poor.

## 1. Introduction

Acute kidney injury (AKI) commonly complicates critical illness and is associated with high mortality and morbidity [1–4]. Early diagnosis of AKI, preferably within 24 hours after ICU admission, is likely pivotal to the development of effective therapies. Traditional biomarkers like creatinine are late indicators of AKI, delaying diagnosis by days [5]. This shortcoming has led to multiple attempts to identify novel biomarkers that can predict the subsequent development of AKI at a much earlier stage [6–8]. However, suggested novel AKI biomarkers, such as neutrophil gelatinase-associated lipocalin (NGAL), have been unreliable in the real world setting of general ICU patients [9].

A recent multicenter international investigation, the Sapphire study, reported the discovery and validation of two G1 cell-cycle arrest biomarkers (CCABs), tissue inhibitor of metalloproteinases (TIMP-2) and insulin-like growth factor binding protein (IGFBP-7) [6]. When combined, these CCABs detected AKI with a high level of accuracy and were excellent in identifying patients at imminent risk of severe AKI [6]. Risk for death, dialysis, or persistent renal dysfunction, that is, major adverse kidney events (MAKE), also increased with increasing test values [6].

Two other studies have assessed the predictive value of [TIMP-2]·[IGFBP7], a multicenter study in a general ICU setting and a single centre study of AKI following cardiac surgery [10, 11]. In both settings, these CCABs predicted AKI with areas under the receiver operating characteristic (ROC) curve of over 0.8. In a separate investigation, done simultaneously but independently of the Sapphire study, IGFBP-7 was identified by proteomics as an early prognostic marker of AKI severity, duration, and mortality [12].

In the present study, we assessed the bedside Nephrocheck Astute 140R Meter (Astute Medical, San Diego, CA), which simultaneously quantifies [TIMP-2]·[IGFBP7] in urine, in a subselected cohort of critically ill patients without evidence of AKI on ICU admission. We compared urinary [TIMP-2]·[IGFBP7] with two other proposed urinary markers of AKI, urinary NGAL and urinary cystatin C. We aimed to investigate the diagnostic value of these markers to detect AKI within 12 to 48 hours of biomarker analysis. Additionally, we intended to explore the potential impact of nonrenal factors with elevated [TIMP-2]·[IGFBP7], NGAL, and cystatin C in urine.

## 2. Methods

This study was approved by the regional ethical review board in Stockholm. Written informed consent was obtained from patients or their next of kin.

### 2.1. Inclusion and Classification of Patients

We studied 138 patients admitted to the general intensive care unit (ICU) at the Karolinska University Hospital, Solna, Sweden, with creatinine clearance >60 mL/min/1.73 m^2^, estimated by the modification of diet in renal disease (MDRD) equation, and an expected length of stay of at least 24 hours. Urine was collected in the morning and in the afternoon until ICU-discharge or initiation of renal replacement therapy (RRT). Patients were included from August 2007 to November 2010. During this time frame, approximately 3500 patients were treated in the ICU. The relatively low number of patients included in this study is mainly explained by the restrictive inclusion criteria. In addition, we were unable to include patients on weekends, nights/on-call hours and during holidays.

The presence or absence of AKI, defined by the Kidney Disease: Improving Global Outcomes (KDIGO) criteria, was recorded on a daily basis [13]. KDIGO grades AKI severity by increase of creatinine from baseline or decrease of urine output. The lowest creatinine level obtained within three months prior to ICU admission was used as baseline for the KDIGO classification. When pre-ICU creatinine was lacking, it was imputed using the MDRD formula, assuming an essentially normal baseline glomerular filtration rate of 75 mL/min/1.73 m^2^. For the purpose of this study we excluded patients who had their first urine sample obtained >48 hours after ICU admission and patients with AKI on the day before and/or on the day when the first study sample was obtained. The primary outcome was development of AKI within 48 hours. Secondary outcomes were development of AKI within 12 hours, within 12 to 24 hours, and within 24 to 48 hours, respectively.

### 2.2. Clinical Data Collection

Acute Physiology and Chronic Health Evaluation (APACHE) II score, demographic data, ICU admission diagnosis, ICU, and 30-day mortality were recorded. Data on comorbid conditions were collected from the hospital-based electronic case-record system. In these electronic journals, prior diagnoses like diabetes, cardiovascular disease, pulmonary disease, gastrointestinal/liver disease, and malignancies are recorded.

Sepsis was defined as a suspected or confirmed infection together with the presence of at least three systemic inflammatory response syndrome (SIRS) criteria [14, 15].

### 2.3. Urine Collection and Biochemical Analyses

After centrifugation at 2000 rpm at 4°C for 10 min, the supernatant urine was stored at −80°C. Samples were analyzed at the Department of Clinical Chemistry, Uppsala University Hospital, Uppsala, Sweden. NGAL was measured by a research enzyme-linked immunosorbent assay (ELISA), as previously described [16, 17]. Monoclonal antibody clones 763 and 764 (Diagnostics Development, Uppsala, Sweden) were used as catching and detecting antibodies, respectively, in the ELISA. Urinary [TIMP2]·[IGFBP7] was quantified by the Nephrocheck point-of-care analyzer (Astute Medical, San Diego, CA), a device that simultaneously measures [TIMP-2] and [IGFBP7] giving a reading based on the multiplication of both biomarkers. Based on the Sapphire study, the manufacturer suggests using >0.3 (ng/mL)^2^/1000 as moderate risk and >2.0 (ng/mL)^2^/1000 as high risk of severe AKI. The test requires 100 *μ*L of urine and 20 minutes to provide results. Urine cystatin C was measured with a particle-enhanced turbidimetric immunoassay on the Architect Ci8200 analyzer (Abbott Laboratories, Abbott Park, IL) with cystatin C reagents from Gentian (Moss, Norway).

### 2.4. Statistical Analysis

Data were analyzed using STATA version 11.2 software (Stata Corporation, College Station, TX, USA). Continuous variables were summarized using median and interquartile range (IQR) and categorical variables as numbers (%). The Kruskal-Wallis test and *χ* ^2^ test were used for comparison between continuous and categorical variables, respectively, across multiple groups. Mann-Whitney test and Fisher's exact test or *χ* ^2^ test were used for two-group comparison between continuous and categorical variables, respectively. The predictive performance for AKI was evaluated by calculating the area under the ROC curve for each urinary biomarker at different time-points. The relationships of [TIMP2]·[IGFBP7], NGAL, and cystatin C with AKI and nonrenal factors were investigated by multivariate linear regression analyses. The following predictor variables were considered: age, gender, APACHE II score, AKI within 48 hours, comorbidities, and admission diagnosis. Predictor variables were included in the multivariate models if they were statistically significant at *P* < 0.1 in the univariate analyses. Two-sided *P* values below 0.05 were considered statistically significant in the final analyses.

## 3. Results

**Figure 1:**
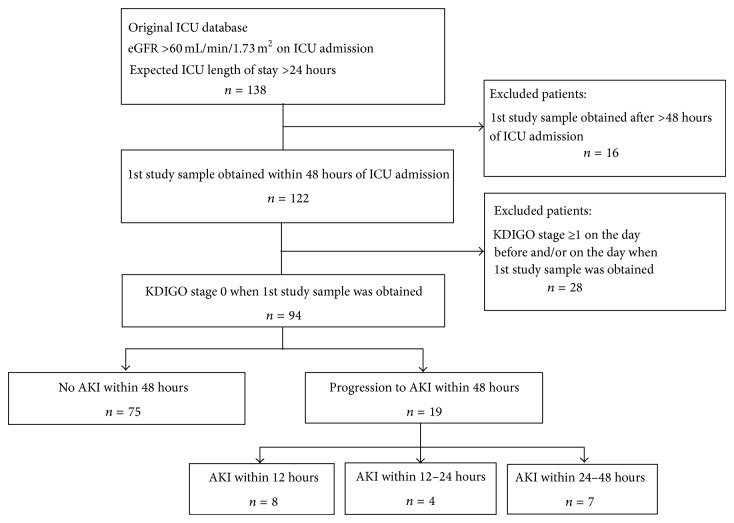
Patient selection.

We screened 138 patients. Of these, 16 patients without available urine sample within 48 hours of ICU admission and 28 patients with AKI on the day of or on the day before the first study sample was obtained were then excluded (Figure 1). Of the remaining 94 patients included in the final analysis, 75 (80%) did not develop AKI within 48 hours whereas 19 patients (20%) did.  Table 1 details the characteristics of the two groups. A true baseline creatinine was available in 73% and 53% of the non-AKI and AKI groups, respectively (*P* = 0.08). APACHE II score and age were higher among patients who developed AKI. Cardiovascular disease was more common among AKI patients, both as an admission diagnosis and as a reported comorbidity. Among the 19 patients who developed AKI within 48 hours, 16 (84%) developed KDIGO stage 1, 2 (11%) developed KDIGO stage 2, and 1 (5%) developed KDIGO stage 3 within this time frame.

[TIMP-2]·[IGFBP7], NGAL, or cystatin C levels on admission did not differ between patients without AKI and patients who developed AKI at different time-points (Table 2). In contrast, peak urinary [TIMP-2]·[IGFBP7] and NGAL levels increased with AKI severity during ICU admission (*P* = 0.002) whereas no such relation was found between urinary cystatin C and AKI severity (*P* = 0.14; Supplementary Table 3in Supplementary Material available online at http://dx.doi.org/10.1155/2015/158658). The predictive properties of the three urinary biomarkers examined are shown in Table 3. In analyzing the cases and controls, we used four differing strategies: prediction of AKI at any time within 48 hours, only within 12 hours, only spanning 12–24 hours, or only during 24–48 hours. All four time-frame strategies failed to produce a ROC area capable of predicting the event.

**Figure 2:**
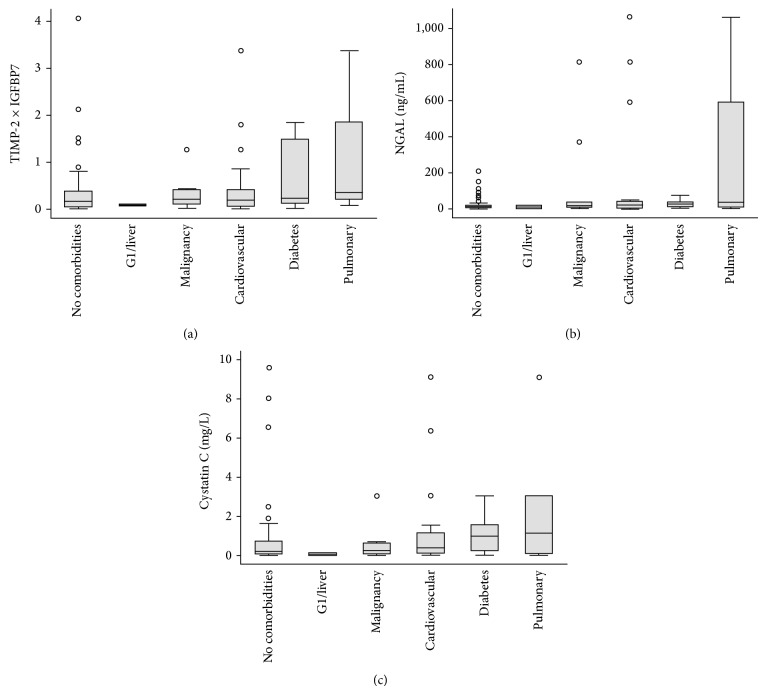
Urinary biomarker levels on ICU admission in non-AKI patients with different comorbidities.

**Figure 3:**
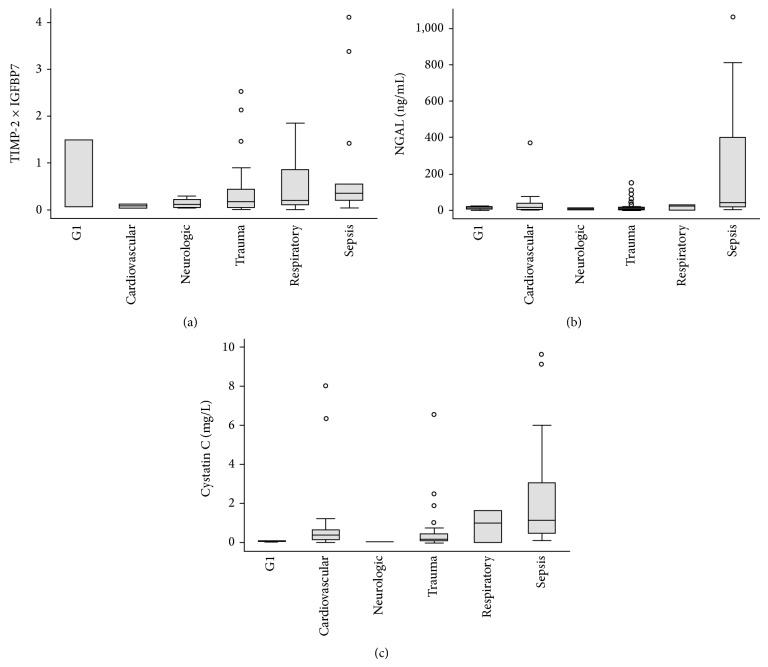
Urinary biomarker levels on ICU admission in non-AKI patients with different admission diagnoses.

We identified biomarker levels associated with comorbidities (Figure 2) and admission diagnoses (Figure 3). Patients with pulmonary disease and diabetes had higher [TIMP-2]·[IGFBP7] levels on admission (Figure 2(a)). For NGAL, a similar pattern was seen in pulmonary disease, but not diabetes (Figure 2(b)). Urinary cystatin C (Figure 2(c)) had a less clear association with comorbidities. In non-AKI patients, individual [TIMP-2]·[IGFBP7] levels were highest in trauma and sepsis patients (Figure 3(a)). NGAL was markedly higher in septic patients, as was cystatin C (Figures 3(b) and 3(c)).

Table 4 and Supplementary Tables 1 and 2 show the unadjusted and adjusted regression coefficients and standard errors relating [TIMP-2]·[IGFBP7], NGAL, and cystatin C to potential predictor variables. Diabetes was independently associated with higher [TIMP-2]·[IGFBP7] (by 4.51 (ng/mL)^2^/1000) whereas AKI was not significantly associated with [TIMP-2]·[IGFBP7] (Table 4). AKI and sepsis were independently associated with higher NGAL (by 525 and 752 ng/mL, resp.; Supplementary Table 1). Only sepsis was independently associated with higher cystatin C (by 1.72 mg/L; Supplementary Table 2).

## 4. Discussion

### 4.1. Key Findings

For the first time, we evaluated cell-cycle arrest biomarkers, analyzed by a commercial point-of-care kit, the Nephrocheck, head to head with urinary NGAL, and urinary cystatin C, in general ICU patients at risk of AKI. Our results failed to show a useful predictive performance for any of the three proposed AKI biomarkers. However, we found an independent association between diabetes and elevated [TIMP-2]·[IGFBP7] levels. In addition, we observed an independent association between sepsis and higher NGAL and cystatin C concentrations in urine.

### 4.2. Relation to Previous Studies

The etiology of AKI is complex and may often be multifactorial, with both ischemic and inflammatory events. Conservation of energy may be an adaptive response of stressed tubular epithelial cells and explain the clinical phenotype of sepsis-induced AKI [18]. One way for cells to save energy is to avoid mitosis. Indeed, it has been shown that renal tubular cells can enter G1 cell-cycle arrest after sepsis [19] and ischemia [20]. Thus, a combination of urinary CCABs like [TIMP-2]·[IGFBP7] has biological plausibility and may have clinical value in these settings. The Sapphire study found that urine [TIMP-2]·[IGFBP7] levels were superior to all previously described early markers of AKI [6]. In a further US-only multicenter study, the high-sensitivity cut-off value of 0.3 (ng/mL)^2^/1000 was tested against clinical nephrologists, who, blinded to the biomarker findings, were asked to determine whether moderate to severe AKI was reached for each patient [10]. Critically ill patients with urinary [TIMP-2]·[IGFBP7] levels > 0.3 (ng/mL)^2^/1000 had seven times the risk for AKI (95% CI 4–22) compared to critically ill patients with a test result below 0.3 [10]. In addition, a study of 50 patients at high risk of AKI following cardiac surgery found that maximum urinary [TIMP-2]·[IGFBP7] concentrations in the 24 hours postoperatively were a sensitive and specific predictor of AKI [11].

Notable differences exist when comparing the original discovery and validation investigations with our study, even though both were done in general ICU cohorts. Moderate to severe AKI (KDIGO stages 2 and 3) on ICU admission was an exclusion criterion in the Sapphire study. However, 32 of 101 (31.7%) had already developed KDIGO 2 or 3 at the time of urine sampling in that study. Moreover, baseline creatinine was significantly higher in patients who developed AKI within 12 hours. In their aggregate, these baseline differences may, to a degree, have inflated the ROC area in the Sapphire study. In contrast, inclusion criteria for our study were stricter. Firstly, we only included patients with a glomerular filtration rate >60 mL/min/1.73 m^2^on admission. Secondly, we excluded patients with KDIGO ≥ 1 at or immediately before first sampling time. We chose this strategy to ensure that no patient had the disease (AKI) when studying its predictors. This methodological distinction is important, as it may explain the relatively low number of AKI cases, the predominance of mild AKI, the scarcity of sepsis as the reason for ICU admission, and the low illness severity scores. More importantly, it may be the main explanation of why all three injury biomarkers failed to predict AKI and why mortality did not differ significantly between AKI and non-AKI patients (Table 1).

The study center is the main trauma referral centre in the region. This explains the relatively higher number of trauma patients in our cohort compared to the Sapphire and Topaz studies. Another difference is that samples were not sent to a central laboratory but were analyzed with a commercial point-of-care analyzer. To our knowledge, this is the first time this has been done in a cohort of general ICU patients.

In contrast to the Sapphire trial, we found an independent association between urinary [TIMP-2]·[IGFBP7] and diabetes, but not with evolving AKI. The association between sepsis and elevated NGAL reported here, however, is not new [21, 22]. Different NGAL forms are released by neutrophils and by epithelial cells from various tissues during systemic inflammation and sepsis [9]. The lack of assays to specifically quantify kidney-specific forms of the NGAL molecule limits its use as an AKI diagnostic tool in undifferentiated ICU patients with sepsis [23]. Interestingly, when NGAL was tested in an emergency department setting, where the prevalence and possibly confounding effects of sepsis were low, the urinary NGAL levels showed excellent predictive values for subsequent AKI [24].

The association between sepsis and urinary cystatin C has also been observed by others [25]. Despite a constant production rate and secretion to the circulation, seemingly unaffected by sepsis [26], urinary cystatin C levels are amplified in septic patients. Impaired tubular reabsorption of middle-sized molecules, such as cystatin C, during sepsis is one likely explanation to this phenomenon [25].

### 4.3. Implications of Study Findings

Adding biomarkers to the clinical assessment of critically ill patients at risk of AKI will be an important step to improve outcomes in such patients. In particular, biomarkers with the ability to predict AKI within 48 hours will be important tools to guide development, validation, and clinical implementation of future strategies to prevent and treat AKI. Our data suggest that when measured by a commercial point-of-care device, the novel CCABs, NGAL, and urinary cystatin C fail to predict evolving AKI in a general cohort of ICU patients. Our findings also suggest that their performance may decrease markedly in general ICU patients, with heterogeneous diagnoses, differing comorbidities, and multiple sources of inflammation.

The associations detected between comorbidities and these biomarkers warrant further exploration. They may partly be explained by concomitant systemic inflammation, which can affect TIMP-2- and IGFBP7-production [18]. The association between elevated CCABs and diabetes might reflect mild- or subclinical, subacute, or chronic diabetes-associated renal injury and creates a major confounder. Further assessment of cell-cycle arrest biomarkers in diabetic patients without AKI seems desirable.

### 4.4. Strengths and Limitations

This study has several strengths. It is prospective in design and detailed in information with daily information on septic state, level of AKI, and biomarker level as well as demographic and comorbidity data. A further strength is that it applies to a general ICU population and, for the first time, used a point-of-care commercial kit for [TIMP-2]·[IGFBP7] measurement. Study limitations include the relatively limited sample size and the inability to measure IGFBP7- and TIMP-2-levels separately. Time from ICU admission to biomarker sampling differed and was sometimes long and was not standardized. However, this is likely to reflect real world practice. Our use of the commercial point-of-care tester could theoretically be a disadvantage; the sandwich immunoassay technique and the fluorescence detection technology might not be as accurate as the central laboratory results. However, it is point-of-care technology that will have to be used in ICU. In addition, we collected urine samples between 2007 and 2010 whereas CCAB analyses were performed during 2013. Such long-term storage may theoretically affect sample concentrations. However, we stored samples at −80°C. It was previously shown that urinary NGAL concentrations remained stable during long-term storage at −80°C [27]. Logically, it is likely that CCABs would remain stable during such similar conditions. Finally, this is a single center study, which limits its external validity.

## 5. Conclusions

In a subset of general ICU patients, the Nephrocheck point-of-care analyzer readings of [TIMP-2]·[IGFBP7] or measurement of the previously suggested urinary biomarkers NGAL and cystatin C did not predict AKI within 12 to 48 hours. Biomarker values were significantly affected by comorbidities even in the absence of AKI. Our findings challenge the robustness and utility of CCABs for the prediction of AKI in general ICU patients.

## Supplementary Material

The Supplementary Material contains more detailed analyses regarding the biomarkers [TIMP-2]•[IGFBP7], NGAL and cystatin C to potential predictor variables.

## Figures and Tables

**Table 1:** Patient characteristics.

	No AKI (*n* = 75)	AKI (*n* = 19)	*P*
Age, years	50 (28, 65)	66 (52, 72)	0.003
Female gender	25 (33%)	3 (16%)	0.17
APACHE II score	15 (11, 19)	19 (13, 24)	0.03
Baseline creatinine, *µ*mol/L	81 (67, 91)	83 (71, 88)	0.64
True baseline creatinine available	55 (73%)	10 (53%)	0.08
Time from ICU admission to first urine sample, hours	11 (5.2, 23)	5.9 (3, 14)	0.13
Comorbidity			
Diabetes	7 (9%)	4 (21%)	0.22
Cardiovascular disease	21 (28%)	11 (58%)	0.01
Pulmonary disease	7 (9%)	1 (5%)	1.0
Gastrointestinal/liver disease	2 (3%)	3 (16%)	0.05
Any malignancy	11 (15%)	3 (16%)	1.0
Admission diagnosis			
Neurologic	5 (5%)	1 (5%)	1.0
Respiratory	16 (21%)	4 (21%)	1.0
Cardiovascular	2 (3%)	5 (26%)	0.003
Trauma	37 (49%)	7 (37%)	0.33
Gastrointestinal	3 (4%)	1 (5%)	1.0
Sepsis	13 (17%)	1 (5%)	0.29
Outcome			
ICU length of stay, days	5 (3, 9.8)	4 (2.9, 7.5)	0.51
ICU mortality	4 (5%)	2 (11%)	0.60
30-day mortality	8 (11%)	4 (21%)	0.25

Values are median (interquartile range) or *n* (%).

**Table 2:** Urinary biomarker levels on admission in non-AKI patients and in patients who develop AKI within 12, 24, and 48 hours.

	No AKI (*n* = 75)	AKI within 12 h	AKI within 12–24 h	AKI within 24–48 h	*P*
		(*n* = 8)	(*n* = 4)	(*n* = 7)	
TIMP-2 × IGFBP7, (g/mL)^2^/1000	0.19 (0.07, 0.51)	0.08 (0.02, 1.30)	0.08 (0.03, 0.68)	0.15 (0.03, 0.40)	0.58
NGAL, ng/mL	10 (3.8, 32)	25 (1.6, 248)	11 (5.5, 30)	7.9 (3.8, 21)	0.95
Cystatin C, mg/L	0.22 (0.09, 0.75)	0.25 (0.01, 4.50)	0.28 (0.15, 0.37)	0.19 (0.07, 0.67)	0.82

Values are median (interquartile range).

**Table 3:** Values for prediction of AKI within 12 to 48 hours.

Urine biomarker	ROC area (95% CI)
Predict AKI within 12 to 48 h (*n* = 94)	
TIMP-2 × IGFBP7	0.40 (0.24–0.57)
NGAL	0.51 (0.36–0.67)
Cystatin C	0.43 (0.27–0.59)

Predict AKI within 12 h (*n* = 83)	
TIMP-2 × IGFBP7	0.41 (0.12–0.71)
NGAL	0.55 (0.27–0.83)
Cystatin C	0.44 (0.12–0.75)

Predict AKI within 12 to 24 h (*n* = 79)	
TIMP-2 × IGFBP7	0.34 (0.00–0.70)
NGAL	0.51 (0.25–0.77)
Cystatin C	0.47 (0.26–0.68)

Predict AKI within 24 to 48 h (*n* = 82)	
TIMP-2 × IGFBP7	0.43 (0.21–0.66)
NGAL	0.47 (0.22–0.72)
Cystatin C	0.41 (0.19–0.63)

**Table 4:** Association between change in level of urinary TIMP-2 × IGFBP7 and clinical factors.

Variable	Not adjusted		Adjusted	
	Coefficient (SE)	*P*	Coefficient (SE)	*P*
Age (per year)	0.03 (0.03)	0.32		
Female gender	−0.85 (1.42)	0.55		
APACHE II score (per point)	0.22 (0.09)	0.02	0.15 (0.09)	0.10
AKI within 48 hours	3.05 (1.59)	0.06	1.86 (1.59)	0.24
Comorbidities				
GI/liver	−1.07 (2.90)	0.71		
Malignancy	−0.77 (1.83)	0.68		
Cardiovascular	1.96 (1.36)	0.15		
Diabetes	5.51 (1.94)	0.006	4.51 (1.96)	0.02
Pulmonary	−0.19 (2.33)	0.94		
Admission diagnosis				
Gastrointestinal	−0.73 (3.23)	0.82		
Cardiovascular	−1.08 (2.48)	0.67		
Neurologic	−1.04 (2.90)	0.72		
Trauma	1.22 (1.30)	0.35		
Respiratory	−0.70 (1.59)	0.66		
Sepsis	−0.23 (1.83)	0.90		

The adjusted regression coefficients, standard errors (SE), and *P* values were estimated from a multiple linear regression model. Multivariate regression includes variables chosen from univariate comparison where *P* < 0.10.
