# Supplementary material for: Assessment of Cell-Cycle Arrest Biomarkers to Predict Early and Delayed Acute Kidney Injury
Source: Dis Markers. 2015 Mar 18;2015:158658. doi: 10.1155/2015/158658 (PMC4381987; doi:10.1155/2015/158658)
Supplement: Supplementary file 1 — The Supplementary Material contains more detailed analyses regarding the biomarkers [TIMP-2]•[IGFBP7], NGAL and cystatin C to potential predictor variables. [file 158658.f1.docx]

Supplementary material

**Supplementary Table 1**. Association between change in level of urinary NGAL and clinical factors.

|  | **Not adjusted** | | **Adjusted** | |
| --- | --- | --- | --- | --- |
| **Variable** | **Coefficient (SE)** | **P** | **Coefficient (SE)** | **P** |
| Age (per year) | 2.21 (3.88) | 0.57 |  |  |
| Female gender | -4.69 (166) | 0.98 |  |  |
| APACHE II score (per point) | -2.99 (11.04) | 0.79 |  |  |
| AKI within 48 hours | 443 (183) | 0.02 | 525 (173) | 0.003 |
| Comorbidities |  |  |  |  |
| GI/liver | -140 (337) | 0.68 |  |  |
| Malignancy | 129 (212) | 0.55 |  |  |
| Cardiovascular | 247 (158) | 0.12 |  |  |
| Diabetes | -99.32 (236) | 0.67 |  |  |
| Pulmonary | 82.55 (271) | 0.76 |  |  |
| Admission diagnosis |  |  |  |  |
| Gastrointestinal | -145 (375) | 0.70 |  |  |
| Cardiovascular | -151 (288) | 0.60 |  |  |
| Neurologic | -146 (337) | 0.67 |  |  |
| Trauma | -229 (151) | 0.13 |  |  |
| Respiratory | -0.53 (185) | 1.00 |  |  |
| Sepsis | 674 (208) | 0.002 | 752 (201) | <0.001 |
| The adjusted regression coefficients, standard errors (SE), and P values were estimated from a multiple linear regression model. Multivariate regression includes variables chosen from univariate comparison where P<0.10. | | | | |

**Supplementary Table 2**. Association between change in level of urinary cystatin C and clinical factors.

|  | **Not adjusted** | | **Adjusted** | |
| --- | --- | --- | --- | --- |
| **Variable** | **Coefficient (SE)** | **P** | **Coefficient (SE)** | **P** |
| Age (per year) | 0.02 (0.01) | 0.14 |  |  |
| Female gender | 0.76 (0.49) | 0.12 |  |  |
| APACHE II score (per point) | 0.02 (0.03) | 0.46 |  |  |
| AKI within 48 hours | 0.10 (0.56) | 0.86 |  |  |
| Comorbidities |  |  |  |  |
| GI/liver | -0.89 (1.00) | 0.38 |  |  |
| Malignancy | 0.02 (0.64) | 0.98 |  |  |
| Cardiovascular | 0.17 (0.48) | 0.72 |  |  |
| Diabetes | 0.51 (0.70) | 0.47 |  |  |
| Pulmonary | 0.96 (0.80) | 0.24 |  |  |
| Admission diagnosis |  |  |  |  |
| Gastrointestinal | -0.42 (1.12) | 0.71 |  |  |
| Cardiovascular | -1.11 (0.85) | 0.20 |  |  |
| Neurologic | -1.04 (1.00) | 0.30 |  |  |
| Trauma | -0.65 (0.45) | 0.15 |  |  |
| Respiratory | 0.53 (0.55) | 0.34 |  |  |
| Sepsis | 1.72 (0.61) | 0.006 | 1.72 (0.61) | 0.006 |
| The adjusted regression coefficients, standard errors (SE), and P values were estimated from a multiple linear regression model. Multivariate regression includes variables chosen from univariate comparison where P<0.10. | | | | |

**Supplementary Table 3**. Peak urinary biomarker levels in relation to worst AKI severity during the entire ICU admission.

|  | No AKI  (n = 61) | KDIGO 1  (n = 25) | KDIGO 2  (n = 5) | KDIGO 3  (n = 3) | P |
| --- | --- | --- | --- | --- | --- |
| TIMP-2 x IGFBP7, (g/mL)^2^/1000 | 0.59 (0.35, 1.40) | 1.20 (0.66, 2.10) | 2.10 (1.00, 3.50) | 2.50 (1.10, 4.60) | 0.002 |
| NGAL, ng/mL | 23 (12, 74) | 44 (21, 149) | 153 (94, 171) | 98 (29, 935) | 0.002 |
| Cystatin C, mg/L | 0.76 (0.32, 3.20) | 1.10 (0.38, 8.20) | 1.60 (0.66, 7.50) | 3.20 (0.01, 27) | 0.14 |
| Values are median (interquartile range). P-values represent non-parametric test for trend across the four groups with increasing AKI severity (No AKI, KDIGO 1, KDIGO 2 and KDIGO 3). | | | | | |
